# Different microRNAs contribute to the protective effect of mesenchymal stem cell-derived microvesicles in LPS induced acute respiratory distress syndrome

**DOI:** 10.22038/IJBMS.2021.56433.12640

**Published:** 2021-12

**Authors:** Xingcai Zhang, Lifang Ye, Guojin Liang, Wan Tang, Lifeng Yao, Changshun Huang

**Affiliations:** 1 Department of Anesthesiology, Ningbo First Hospital, No. 59 Liuting Street, Haishu District, Ningbo 315010, Zhejiang, China; 2 Department of Anesthesiology, Zhujiang Hospital, Southern Medical University, Guangzhou 510280, Guangdong, China

**Keywords:** ARDS, GO and KEGG pathways, Mesenchymal stem cell-derived microvesicles, MicroRNAs, Target

## Abstract

**Objective(s):**

The present study aimed to determine whether bone marrow mesenchymal stem cell-derived microvesicles (MSC MVs) were effective in restoring lung tissue structure, and to assess the potential role of miRNAs in the pathogenesis and progression of acute respiratory distress syndrome (ARDS).

**Materials and Methods:**

ARDS was induced by lipopolysaccharide in male C57BL/6 mice. The degree of lung injury was assessed by histological analysis, lung’s wet weight/body weight, and protein levels in the bronchoalveolar lavage fluid (BALF). Sequencing was performed on the BGISEQ-500 platform. Differentially expressed miRNAs (DEMs) were screened with the DEGseq software. The target genes of DEMs were predicted by iRNAhybrid, miRanda, and TargetScan.

**Results:**

Compared with LPS-injured mice, MSC MVs reduced lung water and total protein levels in the BALF, demonstrating a protective effect. 52 miRNAs were differentially expressed following treatment with MSC MVs in ARDS mice. Among them, miR-532-5p, miR-223-3p, and miR-744-5p were significantly regulated. Gene Ontology (GO) function and Kyoto Encyclopedia of Genes and Genomes (KEGG) pathway analyses revealed the target genes were mainly located in the cell, organelle, and membrane. Furthermore, KEGG pathways such as ErbB, PI3K-Akt, Ras, MAPK, Toll, and Wnt signaling pathways were the most significant pathways enriched by the target genes.

**Conclusion:**

MSC MVs treatment was involved in alleviating lung injury and promoting lung tissue repair by dysregulated miRNAs.

## Introduction

The 2019 novel coronavirus (2019-nCoV) is still spreading rapidly around the world, and the main cause of death in patients remains acute respiratory distress syndrome (ARDS) (1). ARDS is a severe form of hypoxemic respiratory failure, with acute diffuse lung injury, characterized by inflammation leading to increased pulmonary vascular permeability and loss of aerated lung tissue (2). Although a large number of studies have revealed the pathophysiological basis of ARDS, clinically available drugs for its treatment have not reduced mortality, long-term mechanical ventilation time, and ICU hospitalization time in ARDS patients (3). The treatment options for ARDS are still limited. The high mortality rate of ~40% in the United States reflects the lack of efficient medical countermeasures for ARDS (4, 5). Hence, current efforts aimed to develop an effective treatment, and promoting lung tissue repair was the key to improving survival in patients with ARDS.

Because of their immunomodulatory capabilities, mesenchymal stem cells (MSCs) have been widely studied in animal models and clinically. A recent phase IIa clinical trial of MSCs for the treatment of ARDS confirmed a considerable improvement in cell viability from 36% to 85% (6). Interestingly, instead of cell transplantation, MSCs provided beneficial effects through paracrine mechanisms, including secretion of extracellular microvesicles (MVs) (7). Compared to MSCs, MVs have several advantages for use as therapeutic agents. For example, they lack self-replicating capabilities and have no risk of ectopic differentiation, tumor formation, genetic instability, and cellular rejection (8). As a potential “cell-free” therapy, MSC MVs reduced inflammation, promoted healing, and improved organ function (7).

Almost all biological processes, including development, hemostasis, and inflammation, are regulated by miRNAs (2). Currently, miRNAs have been identified as potential therapeutic targets in many diseases such as liver disease, heart disease, and cancer (9-11). Studies have demonstrated that different miRNAs regulated inflammatory reactions and tissue repair mechanisms in ALI/ARDS, which was accompanied by an increase of pro-inflammatory cytokines and a decrease of anti-inflammatory molecules (12-14).

Growing evidence indicated that MSC MVs were involved in protection against lung injury. However, how MSC MVs regulate miRNAs and participate in the pathogenesis and development of ARDS remains unknown. Hence, a successful mouse ARDS model was established in this study, and miRNA expression was profiled following treatment with MSC-MVs, with the aim to determine the potential therapeutic role of MSC MVs in regulating miRNAs in the pathogenesis and development of ARDS. The findings provided new insights into the therapeutic strategy and mechanism of ARDS. 

## Materials and Methods

***Mesenchymal stem cells***

MSCs were purchased from Sciencell (https://www.sciencellonline.com). Cells were routinely identified and met the standard of the International Society of Cellular Therapy. MSCs, with a total passage number of 8, were used in these experiments. MSC MVs were obtained from the supernatant of MSCs as described previously (15).

***Isolation of MSC MVs***

MSCs were cultured in MSC medium (DMEM with 10% fetal bovine serum and penicillin/streptomycin) until confluency in P75 flasks and serum-starved for 48 hr in fresh conditioned medium (α-MEM with 0.5% BSA, 2 mmol/l L-Glutamine, and 1% penicillin/streptomycin). To isolate the MVs, the conditioned medium of MSCs was centrifuged at 3,000 rpm for 20 min to remove cellular debris, then at 100,000g (Beckman Coulter; https://www.beckmancoulter.com/wsrportal/wsr/industrial/products/centrifugation/optima-l-100-xp/index.htm) to pellet the MVs for 1 hr at 48 ^°^C. The pellet was washed with phosphate-buffered saline (PBS) and submitted to the second ultracentrifugation. MSC MVs were resuspended according to the final cell count of MSCs after 48 hr of serum starvation (10 µl MVs per 1*10^6^ cells) and stored at -80 ^°^C until further use (15).

***Identification of MSC MVs***

The isolated MSC MVs were observed under a scanning electron microscope to capture their structure. MSC MVs were fixed with 3% (w/v) Karnovsky fixative for 2 hr at 48 ^°^C. The monolayers were post-fixed for 2 hr with 1% veronal buffered osmic acid and dehydrated in graded ethanol and/or propylene oxide. Cell preparations were then embedded in Epon or Araldite resin cured at 60 ^°^C. Thin sections were contrasted with saturated aqueous uranyl acetate and Reynolds lead citrate. The sections were then imaged with a JEOL 1200 EX transmission electron microscope operating at 80 kV (16). MVs were also assessed for diameter distribution by flow cytometry. Moreover, MVs were evaluated by Western blot to identify specific markers such as CD63 and TSG101.

***ARDS induced by lipopolysaccharide in mice***

Male C57BL/6 mice (8-10 weeks old, n=6) were assessed in all experiments. The mice were purchased from Charles River. All experimental protocols were approved by the Institutional Animal Care and Use Committee of Ningbo University. Mice were first anesthetized with chloral hydrate. ARDS was induced by intra-tracheal (IT) instillation of a non-lethal dose of LPS from *Escherichia coli* (Sigma) at 5 mg/kg (17).

***In vivo experiments***

Mice were first anesthetized with chloral hydrate at 4% intraperitoneally. Different groups were given simultaneously while ARDS was initiated. The various groups included: (a) MSC MVs (100 µl), IT treatment group; (b) LPS (positive control), PBS (100 µl) treatment group; (c) control (negative control), no LPS induction or treatment, with operations similar to the other groups. After 24 hr, and in separate experiments, BALF samples and lungs were collected from mice to assess protein levels and histology, respectively.

***Lung wet weight/body weight and protein levels in BALF***

After the mice were euthanized and weighed, the lungs were extracted and weighed. BALF samples were obtained from mice at 24 hr after LPS-induced ARDS. Total protein concentration was measured in the BALF by the BCA method (BCA, lot: P0010, http://beyotime.com).

***Histology***

Lung samples from LPS-injured mice with or without treatment with MSC MVs were carefully excised at 24 hr and fixed with 4% paraformaldehyde. After fixation, lung samples were embedded in paraffin, cut into 5 μm sections, and stained with H&E. Meanwhile, part of the lung tissue excised was immediately stored in liquid nitrogen for further microRNA analysis.

***Identification of miRNAs***

Mice (n=3) were randomly selected from each group and sequenced on the BGISEQ-500 platform. With Bowtie (bowtiebio.sourceforge.net), cleaned miRNA sequencing reads were aligned to the human genome (GRCh38.p7 assembly) based on the Genome human UCSC reference annotation. With miRDeep2 (https://www.mdc-berlin.de/8551903/en/), the transcription abundance of miRNAs was determined. DEMs between the treatment and non-treatment groups were screened using the DEGseq software. The miRNA sequencing data generated in this paper is available in BioProject (PRJNA662018). For DEMs in the LPS+MV group versus LPS group, the threshold was defined as Q value<0.001 and |log2FC|>1.

***Identification of DEMs’ target genes***

To improve the reliability of the predicted target genes, the target genes of DEMs were predicted by three algorithms, including iRNAhybrid (18), miRanda (19), and TargetScan (20), and filtered by the corresponding filter conditions such as free energy and score value. Genes that were simultaneously predicted by all three databases were identified as the target genes of DEMs.

***Functional annotation of DEMs***

Enrichment analysis was performed to assess whether a particular gene set was significantly enriched in a metabolic pathway, molecular function, or biological process. To further examine the biological functions of the target genes of DEMs, GO and KEGG pathway enrichment analyses of these DEMs between the LPS+MSC MVs and LPS groups were performed with the R package “hphyper”. Adjusted *P*-values (Q values) for each pathway were obtained by FDR correction. Q value<0.05 was defined as the criterion for statistical significance.

***Statistical analysis***

Group pair comparisons were performed by unpaired t-test. For multiple group comparisons, ANOVA with Bonferroni correction was performed. *P*<0.05 was considered statistically significant. Analyses were performed with GraphPad Prism6 and Image. The severity of lung injury was semi-quantified with the lung injury score system by two investigators blinded to grouping.

## Results

***Characterization of MSC MVs***

**Figure 1:**
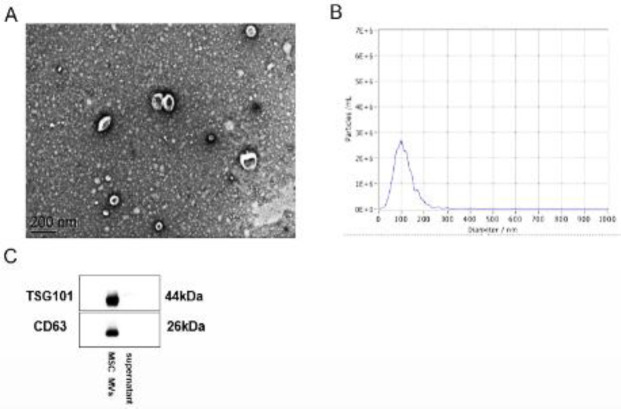
Isolation and characterization of MSC MVs. A. Transmission electron micrographs of MSC MVs. Scale bar, 200 nm. B. Size distribution profile of MSC MVs. C. Western blot analysis of MSC MV markers (CD63 and TSG101) in exosome preparations

MSC MVs were characterized by electron microscopy, NanoSight analysis and Western blot, respectively. Scanning electron microscopy showed that the isolation technique yielded a homogeneous population of spheroid particles (Figure 1A). Then, Nanoflow detection showed that the diameters of MSC MVs were mostly around 67.72 nm, with a concentration of 1.16*10^11 ^particles per ml (Figure 1B). The main protein composition of MSC MVs was analyzed by Western blot, which revealed that MSC MVs expressed membrane proteins commonly found in exosomes (CD63) and an endosome-associated protein (TSG101) (Figure 1C). These results confirmed that MVs derived from MSCs had the typical features of EVs in terms of size and protein marker profile. 

***MSC MVs alleviated LPS-induced lung inflammation and injury***

**Figure 2:**
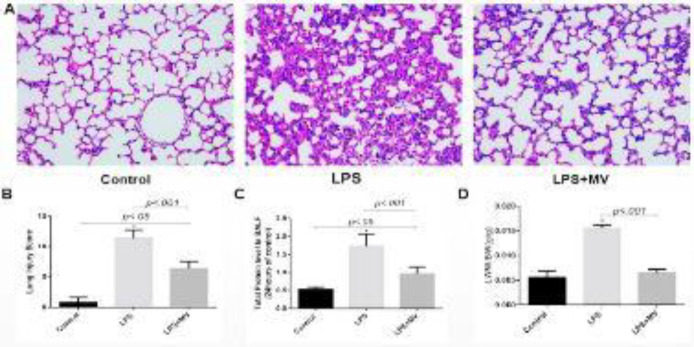
MSC MVs alleviated LPS-induced lung injury in mice. A. Representative histological images of lung samples in different groups; original magnification, ×200. B. Lung injury scores in different groups. C. Lung permeability was assessed by measuring total BALF protein levels and LWW/BW ratios. Values were mean±SEM (n=6)

To establish a successful model of ARDS, we chose 24 hr after vehicle or LPS instillation for assessment of lung inflammation and injury. Compared with the control group, there was increased alveolar septal thickening and elevated leukocyte infiltration in the alveolar and interstitial spaces in the LPS group. Compared with the LPS group, MSC MVs obviously relieved LPS-induced lung injury (Figure 2A). Lung injury scores were significantly increased in the LPS group compared with the LPS+MSC MVs group (Figure 2B). Pulmonary microvascular EC plays a pivotal role in the pulmonary inflammatory response. To address whether MSC MVs modulated pulmonary vascular EC barrier functions *in vivo*, BALF protein concentration and LWW/BW ratio were analyzed in LPS-induced ARDS mice. Total protein amounts were higher in the LPS group compared with the control group. Surprisingly, total protein concentrations were decreased in the LPS+MSC MVs group (Figure 2C). LWW/BW ratios (Figure 2D) in ARDS mice were significantly reduced by treatment with MSC MVs. 

***Quality of the sequencing process***

**Figure 3:**
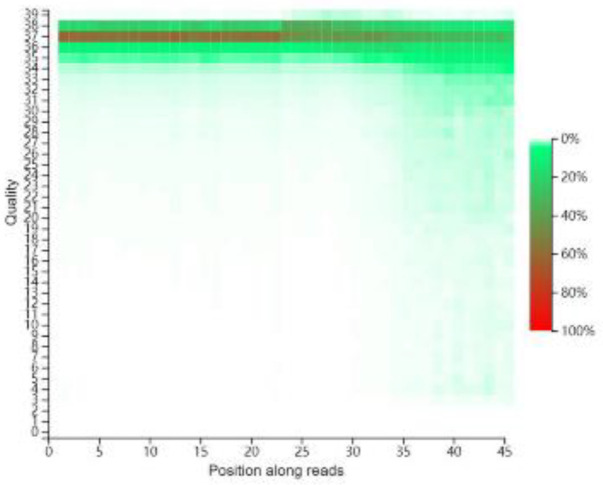
X-axis represents the positions of bases in the reads, and the Y-axis represented the base quality. Each point represented the total number of bases reaching a certain quality at this position

SOAPnuke was used to control sample quality (https://github.com/BGI-flexlab/SOAPnuke). The base quality distribution reflected the accuracy of sequencing reads. The sequencer, sequencing reagents, and sample quality could all affect the quality of bases. The quality of the first few bases in the reads was not high, because random primers could not bind to the RNA template well during reverse transcription; as the length of sequencing increased, the ratio of high-quality bases increased. A low proportion of low-quality (Quality<20) bases indicated good sequencing quality (Figure 3).

***Identification of differentially expressed microRNAs***

**Figure 4:**
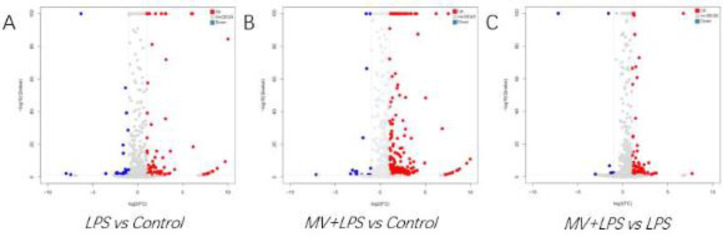
Volcano plots of DEMs in different groups. (A) 72 miRNAs were differentially expressed in ARDS mice. (C) 52 miRNAs were differentially expressed following treatment with MSCs MV in ARDS mice. Red, blue, and gray dots represent up-regulated, down-regulated, and non-differentially expressed miRNAs, respectively

Using a significance threshold of *P*<0.05, 72 miRNAs with differential expression between LPS and control groups were found, including 62 up-regulated and 10 down-regulated miRNAs. Among them, miR-146a-3p and miR-155-5p were significantly up-regulated (Figure 4A). Sequencing analysis revealed that 52 miRNAs were differentially expressed following treatment with MSC MVs in mice with ARDS. Of these 52 miRNAs, 49 were up-regulated and 3 were down-regulated. Among them, miR-532-5p, miR-223-3p, and miR-744-5p were significantly regulated (Figure 4C).

***Function and pathway analyses***

**Figure 5:**
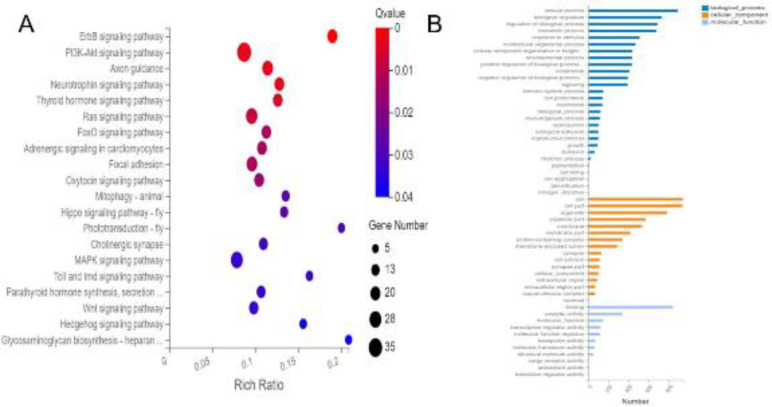
Enriched GO terms and KEGG pathways of DEMs in the LPS with MSC MVs group. A. KEGG pathways, where the color and size of the circle indicate the significance level (Q value) and the number of target genes involved, respectively. The x-axis shows the enrichment ratio and the y-axis shows KEGG pathways. B. GO classification, where the x- and y-axes represent the number of genes and the functional classification of GO, respectively

GO function and KEGG pathway analyses were performed to explore the biological functions of the identified target genes. Compared with the LPS group, genes were significantly enriched in cellular process, biological regulation, and metabolic process; in addition, the target genes were mainly located in the cell, organelle, and membrane (Figure 5B). Furthermore, KEGG pathways such as the ErbB, PI3K-Akt, Ras, MAPK, Toll, and Wnt signaling pathways were the most significant pathways enriched by the target genes in the LPS+MSC MVs group (Figure 5A).

***MiRNA-gene regulation network construction and analysis***

**Figure 6.**
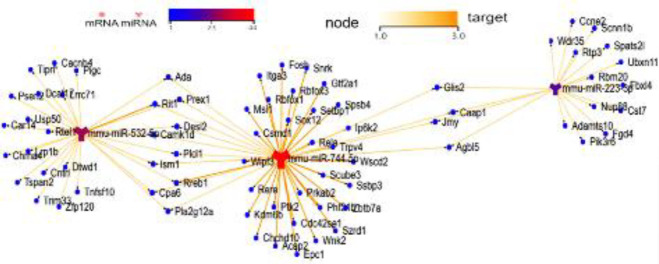
DEmiRNA-mRNA interaction network. Interaction network between significantly down-regulated DEMs and up-regulated DEMs in the LPS with MSC MVs group. Compared with the LPS group, miR532-5p and miR223-3p were up-regulated, while miR744-55 was down-regulated

In order to investigate the functions of differentially expressed miRNAs, we used three databases, including miRanda, miRTarBase, and TargetScan, to identify the target genes of lung tissue that were significantly up-regulated or down-regulated. Then, the confirmed DEmiRNA-DEmRNA interactions were further used for building a miRNA-gene regulation network (Figure 6).

## Discussion

Studies have investigated the potential role of miRNAs in the pathogenesis of acute lung injury (ALI)/ARDS. MiRNAs are a class of highly conserved, noncoding single-stranded RNA molecules of approximately 18–22 nucleotides (21). Almost all biological processes, including development, hemostasis, and inflammation, were regulated by miRNAs. Multiple reports that analyzed the expression of miRNAs in pulmonary diseases were based on preclinical studies. However, the underlying mechanisms are not fully understood.

In this work, we found that compared with the control group, 72 miRNAs were differentially expressed in ARDS mice, including 62 up-regulated and 10 down-regulated. Among them, miR-146a-3p and miR-155-5p were significantly up-regulated. Previous studies confirmed that down-regulation of microRNA-146a-3p attenuated lipopolysaccharide-induced acute lung injury via SIRT1 up-regulation and NF-κB pathway regulation (22). As a key mediator of septic lung injury, serum exosomes from ALI mice delivered miR-155 to macrophages, stimulated nuclear factor κB (NF-κB) activation, and induced the production of tumor necrosis factor-alpha (TNF-α) and interleukin (IL)-6 (23).

In most applications, MSCs were captured in the lungs and disappeared after a few days, with only a small proportion recovered at the injury site after intravenous injection (24, 25). It has been proposed that the immunomodulatory and regenerative effects of mesenchymal stem/stromal cells (MSCs) were mainly mediated by soluble paracrine factors and MSC-derived extracellular vesicles (MSC EVs)(26). Recent studies suggested that MSC EVs might serve as a novel and cell-free alternative to whole-cell therapies. Extracellular vesicle (EV) is a general term for membrane surrounded vesicles released by cells. According to size and biogenetic mechanisms, EVs are typically classified into three subtypes, including exosomes (50–150 nm), MVs (100–1000 nm), and apoptotic bodies (500–5000 nm)(27). Since no specific exosome and microvesicle markers have been identified, vesicles can only be fractioned according to size and/or density, regardless of their origin (8). 

Previous studies confirmed that MSC MVs transfer depolarized mitochondria to macrophages, increase macrophage bioenergetics, and modulate TLR signaling and cytokine release in macrophages (28). *In vitro*, MSC MVs enhanced the junctional integrity of injured endothelial cells, decreased lung pathology injury, modulated cytoskeletal signaling in endothelial cells, and attenuated lung vascular permeability (29, 30). *In vivo*, MSC MVs increased alveolar fluid clearance, reduced protein permeability and inflammation, and increased antimicrobial effects in the human lung model of bacterial pneumonia (26). In this study, the therapeutic effects of MSC MVs were examined in an LPS-induced ARDS mouse model. Histopathological examination indicated that lung injury was less prominent in the ARDS group treated with MSC MVs compared with the ARDS group. Compared with the LPS group, MSC MVs obviously relieved LPS-induced lung injury. Lung injury scores were significantly increased in the LPS group compared with the LPS+MSC MVs group. Total protein levels in the LPS group were higher than control values. Surprisingly, total protein concentrations in the BALF were decreased in the MSC MVs treatment group. LWW/BW ratios in mice were significantly reduced by treatment with MSC MVs. These results fully confirmed that MSC MVs alleviated LPS-induced lung inflammation and injury. Meanwhile, our data revealed 52 miRNAs were differentially expressed following treatment with MSC MVs in ARDS mice. Of the 52 miRNAs, 49 were up-regulated and 3 were down-regulated. Among them, miR532-5p, miR223-3p, and miR744-5p were significantly regulated. Previous investigations performing miRNA profiling in various inflammatory settings revealed that miR-223 was among the top differentially expressed miRNAs; indeed, the expression of miR-223 in an inflammatory model of GBS-induced murine pneumonia was rapidly induced at very early time points (3 to 6 hr post-infection)(31). MiR223 levels were significantly decreased in patients with non-surviving sepsis compared with surviving sepsis cases (32). Exosomes from MSCs overexpressing miR-223-3p suppressed the production of pro-inflammatory factors and promoted the secretion of anti-inflammatory factors, and attenuated cerebral ischemia/reperfusion injury by inhibiting microglial M1 polarization mediated pro-inflammatory response (33). Overexpression of miR-532-5p restrains the elevation of inducible NOS, IL-6, IL-1 β, TNF-α, and MCP-1 in LPS-exposed BV2 cells (34). MiR-532-5p could inhibit the IL-1β-induced NF-κB proinflammatory signaling pathway, which suggested that miR-532-5p might be a potential anti-inflammatory mediator attenuating the symptoms of asthma (35). These miRNAs were detected in the present study in the lung tissue, which indicates that our approach provides reliable information regarding the miRNA expression profile.

In order to determine the biological effects of the target genes identified, we performed GO and KEGG pathway analyses of these genes in the lung after treatment with LPS or LPS with MSC MVs. The results showed the target genes were likely associated with ErbB, PI3K-Akt, Ras, MAPK, Toll, and Wnt signaling pathways. Both PI3K/Akt and MAPK intracellular cascades can be initiated by LPS-TRL4 receptor activation, leading to gene transcription and proinflammatory cytokine production; in particular, p38 MAPK plays a significant role in lung inflammation and cell apoptosis and is activated by LPS (36-38). The Toll-like receptor (TLR)4 receptor complex, TLR4/MD-2, plays an important role in the inflammatory response against lipopolysaccharide, and modifications in surface TLR4 expression via overexpression of Ras-related protein Rab10 in macrophages exacerbates LPS-induced lung injury (39). Therapeutic manipulation of Wnt signaling in endogenous stem cells may be exploited for tissue renewal and regeneration during early ARDS(40). GO classification demonstrated that the target genes were primarily related to cellular processes, biological regulation, and metabolic processes. Moreover, the target genes were mainly located in the cell, organelle, also membrane.

A single miRNA can have multiple target mRNAs, and different sets of miRNAs are expressed in different cell types and tissues, with multiple functions in the development of many biological processes. In this study, a regulatory network of significantly up-regulated and down-regulated miRNA genes was built. However, the specific targeting relationships of these miRNA-mRNA sets need to be further verified. 

This study had a few limitations. First, the small sample size might decrease the statistical power. In addition, the dysregulated miRNAs following LPS injection or MSC MVs infusion were not quantified by reverse transcription-quantitative polymerase chain reaction. Furthermore, we constructed a signaling pathway diagram based on sequencing results, but no further experiments were conducted to verify whether the effectors were involved in lung injury repair. Finally, MSC MVs were only administered via the tail vein to explore their protective effect on the lung. However, whether administration via the airway would be better remains unknown. Therefore, different treatment routes for MSC MVs deserved further investigation. In spite of these limitations, this study demonstrated that dysregulated miRNAs were involved in lung injury alleviation and lung tissue repair promotion in mice following treatment with MSC MVs.

## Conclusion

In summary, MSC MVs attenuated lung injury via miRNA dysregulation, indicating the potential of miRNAs as therapeutic targets. Further studies are required to elucidate the putative targets of the identified dysregulated miRNAs.

## Authors’ Contributions

XCZ Conceived and designed research. XCZ and LFY Performed experiments; XCZ Wrote the manuscript; GJL Analyzed data; WT Interpreted results of experiments; LFY Prepared figures; CSH Edited and revised manuscript; CSH Approved final version of manuscript.

## Conflicts of Interest

The authors declare no conflicts of interest.
